# *OsIAGT1* Is a Glucosyltransferase Gene Involved in the Glucose Conjugation of Auxins in Rice

**DOI:** 10.1186/s12284-019-0357-z

**Published:** 2019-12-18

**Authors:** Qian Liu, Ting-Ting Chen, Dong-Wang Xiao, Shu-Man Zhao, Ji-Shan Lin, Ting Wang, Yan-Jie Li, Bing-Kai Hou

**Affiliations:** 1 The Key Lab of Plant Development and Environment Adaptation Biology, Ministry of Education of China, School of Life Sciences, Shandong University, Qingdao, 266237 China; 2 Present Address: Center for Genomics and Biotechnology, Haixia Institute of Science and Technology, Fujian Agriculture and Forestry University, Fuzhou, 350002 China

**Keywords:** *Oryza sativa*, Auxin, glucosyltransferase, Enzyme activity, glucose ester conjugates

## Abstract

**Background:**

In cereal crop rice, auxin is known as an important class of plant hormone that regulates a plethora of plant growth and development. Glycosylation of auxin is known to be one of the important mechanisms mediating auxin homeostasis. However, the relevant auxin glucosyltransferase (GT) in rice still remains largely unknown.

**Results:**

In this study, using known auxin glucosyltransferases from other species as queries, twelve putative auxin UDP-glycosyltransferase (UGT) genes were cloned from rice and the one showing highest sequence similarity, named as *OsIAGT1*, was expressed as recombinant protein. In vitro enzymatic analysis showed that recombinant OsIAGT1 was capable of catalyzing glucosylation of IAA, IBA and other auxin analogs, and that OsIAGT1 is quite tolerant to a broad range of reaction conditions with peak activity at 30 °С and pH 8.0. OsIAGT1 showed favorite activity towards native auxins over artificially synthesized ones. Further study indicated that expression of *OsIAGT1* can be upregulated by auxin in rice, and with *OsIAGT1* overexpressing lines we confirmed that OsIAGT1 is indeed able to glucosylate IAA in vivo. Consistently, ectopic expression of *OsIAGT1* leads to declined endogenous IAA content, as well as upregulated auxin synthesis genes and reduced expression of auxin-responsive genes, which likely leads to the reduced plant stature and root length in *OsIAGT1* overexpression lines.

**Conclusion:**

Our result indicated that OsIAGT1 plays an important role in mediating auxin homeostasis by catalyzing auxin glucosylation, and by which OsIAGT1 regulates growth and development in rice.

## Background

Auxins are a class of plant hormones that plays a vital role in many aspects of plant growth and development, including embryonic apical-basal axis formation, vascular development, postembryonic organogenesis, plant tropism, fruit development, stress response, senescence and apical dominance (Sundberg and Ostergaard 2009; Grunewald and Friml 2010; Scarpella et al. 2010). Structurally, auxins are known to have an aromatic ring with a side chain containing a carboxyl group, and the most abundant endogenous auxin is indole-3-acetic acid (IAA). It was shown that several types of alkylated derivatives of IAA also show strong bioactivity (Nigović et al. 2000). In addition to IAA, there are also other three endogenous auxin forms, namely 4-chloroindole-3-acetic acid (4-Cl-IAA), indole-3-butyric acid (IBA) and phenylacetic acid (PAA) (Simon and Petrášek 2011; Liu et al. 2012). In the long process of auxin function analysis, several other non-endogenous compounds were found to show auxin activity, classified as synthetic auxins, e.g. 2,4-dichlorophenoxyacetic acid (2,4-D) and naphthalene-1-acetic acid (NAA).

The auxin responses in plants are strictly dose-dependent, excess auxin often causes inhibitory effects. Therefore endogenous auxin homeostasis is always tightly controlled by various aspects, including biosynthesis, degradation, transport, and conjugate modification (Ludwig-Müller 2011). Several major auxin conjugates had been identified in higher plants, including ester-linked IAA-sugar conjugates, amide-linked IAA-amino acid conjugates, and methyl ester of IAA (Jackson et al. 2001; Qin et al. 2005; Ludwig-Müller 2011; Casanova-Sáez and Voß 2019). Auxin conjugates are usually considered inactive and can be hydrolyzed to active forms. Interestingly, the composition of IAA conjugates varies in different plant species, e. g., the major conjugate form in maize kernels is ester-linked sugars (Bandurski et al. 1995), whereas Arabidopsis and many other dicots mainly store amide-linked amino acid IAA conjugates (Bajguz and Piotrowska 2009).

Glycosyltransferases (GTs) are responsible for transferring sugar moieties to a wide range of receptor molecules, including plant hormone auxin. These enzymes can be classified into at least 107 sub-families so far, and the largest sub-family is family 1 glycosyltransferases (GT1), also known as UDP-glycosyltransferases (UGTs) (Yonekura-Sakakibara and Hanada 2011). In maize, *iaglu* was the first UGT identified to be responsible for the conjugation of IAA with glucose (Szerszen et al. 1994). In Arabidopsis, several UGTs were found to catalyze auxin glycosylation. UGT84B1 showed high activity towards IAA (Jackson et al. 2001), and UGT74E2 is responsible for IBA glycosylation (Tognetti et al. 2010), while UGT74D1 shows activity toward several auxins, including IAA and IBA (Jin et al. 2013). A recent study indicated that the endogenous function of UGT74D1 is to catalyze 2-oxindole-3-acetic acid (OxIAA) to OxIAA-glucoside (OxIAA-Glc) (Tanaka et al. 2014). These auxin UGTs also play an important role in plant growth and development. Perturbation of UGT84B1, UGT74E2 and UGT74D1 leads to altered shoot architecture and stress responses. On the other hand, as an inactive form, IAA sugar conjugates were considered to play a role in auxin storage. For example, IAA ester conjugates were found to be widely exists in maize immature kernels, and can be hydrolyzed to free IAAs (Jakubowska and Kowalczyk 2005), implying the role of glycosylation modification in maintaining auxin homeostasis.

As one of the major food crops, rice is extensively studied as model plant due to its relatively compact genome (Brozynska et al. 2016). Putative cytokinin glucosyltransferases cZOGT1, cZOGT2, and cZOGT3 were shown to regulate shoot stature and leaf senescence (Kudo et al. 2012). However, the study on hormone glycosylation in rice is still limited. In this study, using known auxin UGTs as queries, we cloned twelve putative auxin UGT genes from rice. The rice UGT showing highest similarity with known auxin UGTs of other species was named as OsIAGT1 (LOC_Os03g48740). Enzymatic reaction analysis showed that OsIAGT1 is capable of catalyzing auxin glucosylation both in vitro and in vivo, with a quite broad range of reaction conditions. Moreover, we found that expression of *OsIAGT1* is auxin-induced, and ectopic expression of *OsIAGT1* leads to declined endogenous IAA content, as well as reduced expression of auxin-responsive genes, which caused the reduced plant height and root length in *OsIAGT1* overexpression lines. OsIAGT1 is the same protein to a recently reported UGT in another study, which was designated as OsIAAGLU (Yu et al. 2019). Our results further strengthen the role of OsIAGT1/OsIAAGLU in mediating auxin homeostasis by catalyzing auxin glucosylation and in regulating rice growth and development.

## Results

### Identification of Putative Auxin Glycosyltransferases in Rice

**Fig. 1:**
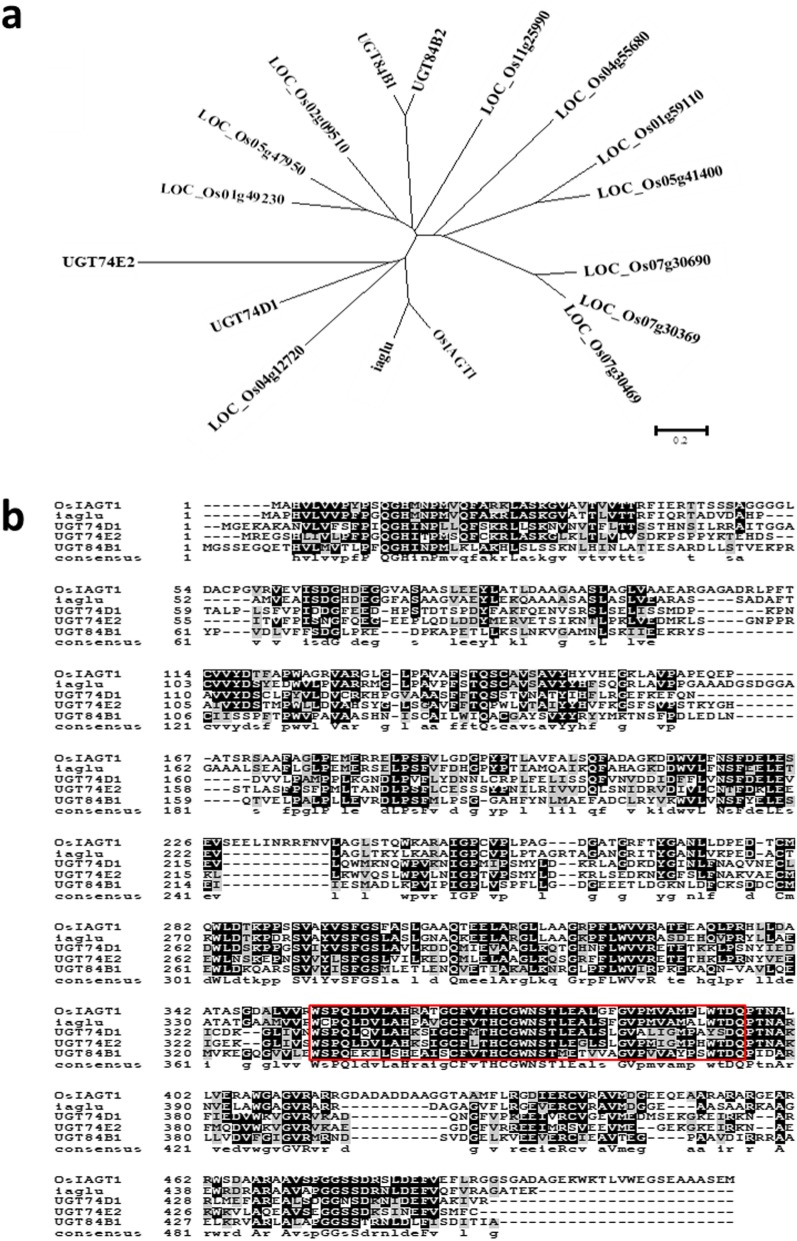
Phylogenetic analysis of OsIAGT1. **a** Phylogenetic analysis of 12 rice genes and other auxin UGTs from maize and Arabidopsis. Rice LOC_Os03g48740 is named OsIAGT1. **b** Alignment of amino acid sequence of OsIAGT1 with other auxin UGTs from maize and Arabidopsis. The C-terminal conserved glycosyltransferase PSPG domain was shown in red box

BLAST searches were performed in open-access rice genome databases using amino acid sequences of maize auxin UGT protein iaglu (Szerszen et al. 1994) and Arabidopsis UGT84B1, UGT84B2, UGT74E2 and UGT74D1 as queries. The result revealed twelve rice nuclear genes, i.e. *LOC_Os01g49230, LOC_Os01g59110, LOC_Os02g09510, LOC_Os03g48740*, *LOC_Os04g12720, LOC_Os04g55680, LOC_Os05g41400, LOC_Os05g47950*, *LOC_Os07g30369, LOC_Os07g30469, LOC_Os07g30690* and *LOC_Os11g25990*, which turned out to be potential auxin UGTs. The complete predicted amino acid sequence for LOC_Os03g48740 shows highest similarity to maize iaglu protein, and was named as OsIAGT1 in this study (Fig. 1a). We then performed multiple alignment using the full amino acid sequences of the rice OsIAGT1, Arabidopsis UGT74D1, UGT74E2 and UGT84B1, and maize iaglu (Fig. 1b). The result showed high similarity and a C-terminal conserved glycosyltransferase domain PSPG box was identified in all the five proteins, suggesting that the rice OsIAGT1 is likely to be homologous with the other four UGTs.

### In Vitro Biochemical Characterization and Kinetics of OsIAGT1 towards Auxins

**Fig. 2:**
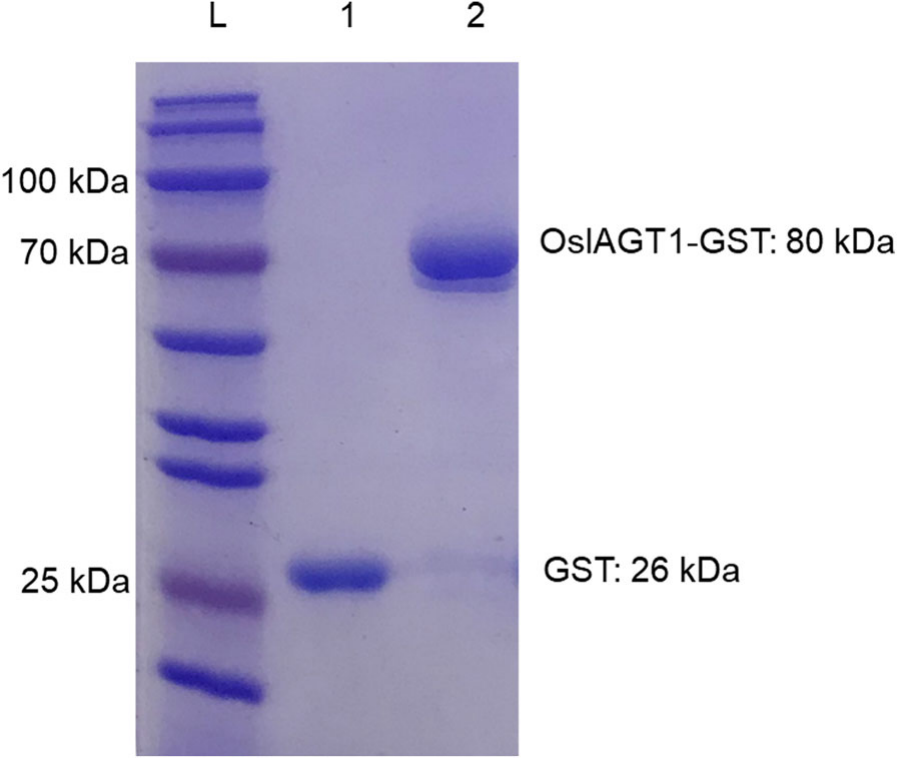
SDS-PAGE analysis of the recombinant GST-OsIAGT1 fusion protein. L, protein molecular weight ladder; 1, GST-tag protein; 2, OsIAGT1 fusion protein

To examine glucosylation activity of the putative OsIAGT1, GST-tagged proteins were expressed in *Escherichia coli* cells, purified by Glutathione Sepharose 4B affinity chromatography. After purification of fusion protein, the protein was subjected to SDS-PAGE analysis. As predicted, the purified OsIAGT1 fusion protein showed a molecular weight of approximately 80 kDa (Fig. 2). Meanwhile, GST-tag protein was used as a negative control. Arabidopsis UGT74D1 was previously demonstrated to have a glucosylating activity toward auxin (Jin et al. 2013), its recombinant protein was purified and used as a positive control in the following enzyme reaction.

**Fig. 3:**
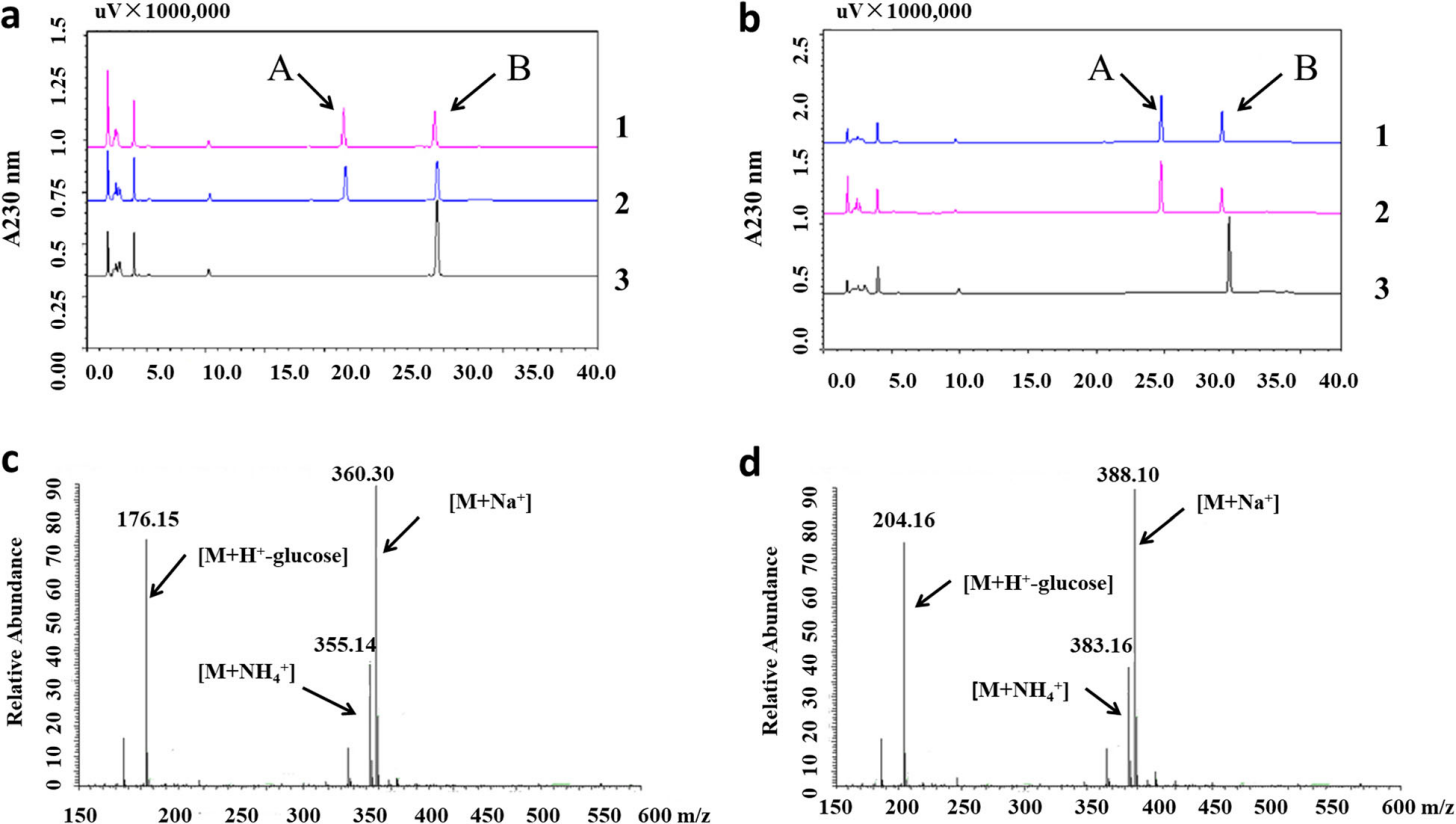
HPLC and LC-MS analysis of reaction products from IAA and IBA catalyzed by OsIAGT1. **a** and **b** HPLC analysis of reaction products from IAA and IBA, respectively. 1, 2, 3 denote the reactions with recombinant protein OsIAGT1, UGT74D1 (positive control), GST (negative control), respectively. “A” denotes the possible products; “B” denotes the substrate IAA or IBA. **c** and **d** LC–MS analysis of reaction products from IAA and IBA, respectively

Using UDP-glucose as the sugar donor, purified recombinant proteins were tested in vitro for the glucosyltransferase activity against IAA and IBA. The HPLC analysis of reaction products and the recognition of new product peaks indicated that recombinant OsIAGT1-GST, similar to the Arabidopsis UGT74D1, might be capable of catalyzing IAA and IBA to form their glucose ester conjugates (IAA-Glc and IBA-Glc) (Fig. 3a and b). In order to verify the identity of reaction products, we conducted a LC-MS analysis. As shown in Fig. 3c and d, in the positive ionization mode, the predicted reaction products of IAA-Glc gave a dominant ions m/z 176.15 (M + H^+^-glucose); m/z 355.14 (M + NH_4_^+^) and m/z 360.30 (M + Na^+^). The predicted reaction products of IBA-Glc gave a dominant ions m/z 204.16 (M + H^+^-glucose); m/z 383.16 (M + NH_4_^+^) and m/z 388.10 (M + Na^+^), fitting well with the molecular weight (MW) of IAA-Glc (MW = 337.00) and IBA-Glc (MW = 365.00), respectively. Therefore, our results indicated that rice OsIAGT1 is likely responsible for catalyzing IAA and IBA glucosylation.

### Biochemical Characterization of OsIAGT1

**Fig. 4:**
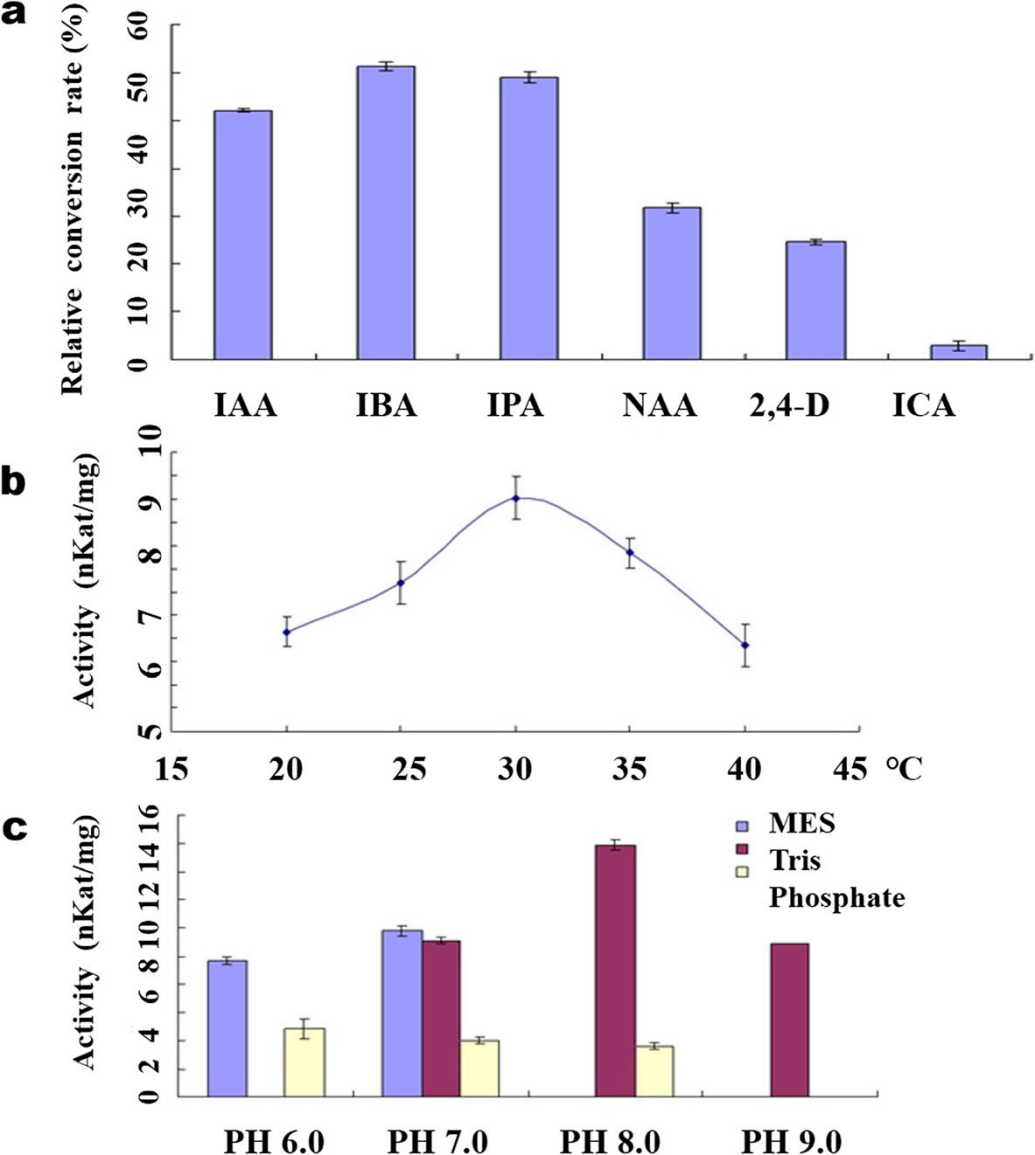
Biochemical characterization of OsIAGT1. **a** Relative activity of OsIAGT1 toward auxins and related substrates. **b** The effect of temperature on the activity of recombinant OsIAGT1 towards IAA (in 50 mM Tris.HCl). **c** The effect of different recombination of buffer and pH on the activity of recombinant OsIAGT1 towards IAA (at 30 °С). Values are means ±SD (*n* ≥ 3)

Besides IAA and IBA, we also tested the activity of OsIAGT1 towards other forms of auxins, including NAA, 2,4-D, Indole-3-propionic acid (IPA) and indole-3-carboxylic acid (ICA). The activity of OsIAGT1 towards these auxins was also determined with HPLC analysis and LC-MS analysis. Similar to what had been observed with IAA and IBA, our result confirmed that OsIAGT1 was also able to catalyze the glycosylation of IPA, NAA, 2,4-D and ICA (Additional file 2: Figure S1). The observed ions information of reaction products of IPA, NAA, 2,4-D and ICA with recombinant GST-OsIAGT1 in LC-MS analysis were as follows: in the positive ionization mode, the predicted products of IPA-glucose ester (IPA-Glc) gave a dominant ions m/z 352 (M + H^+^); m/z 374 (M + Na^+^). The predicted reaction products of NAA-Glc gave a dominant ions m/z 349 (M + H^+^); m/z 366 (M + NH_4_^+^) and m/z 371 (M + Na^+^). The predicted reaction products of 2,4-D-Glc gave a dominant ions m/z 383 (M + H^+^); m/z 401 (M + NH_4_^+^). The predicted reaction products of ICA-Glc gave a dominant ions m/z 324 (M + H^+^) and m/z 346 (M + Na^+^). All the ions information fits well with their MW of these four auxins. To compare the catalytic efficiency of OsIAGT1 towards these different forms of auxins, their relative conversion rates were calculated based on the relative reduction rates of these auxins after the in vitro enzymatic reaction. Interestingly, OsIAGT1 shows relatively higher activity towards IAA, IBA and IPA, compared to NAA, 2,4-D and ICA (Fig. 4a).

Next, using IAA as substrate, the optimal reaction conditions of OsIAGT1 were tested including temperature, pH and buffer type (Fig. 4b and c). The result indicated that OsIAGT1 is quite tolerant to different reaction conditions, which shows enzymatic activity under reaction temperature ranging from 20 °С to 40 °С with peak activity at 30 °С, and pH ranging between 6.0 ~ 9.0 with peak activity with pH 8.0 Tris buffer.

### *OsIAGT1* Expression Pattern and Induced Upregulation by Auxin

**Fig. 5:**
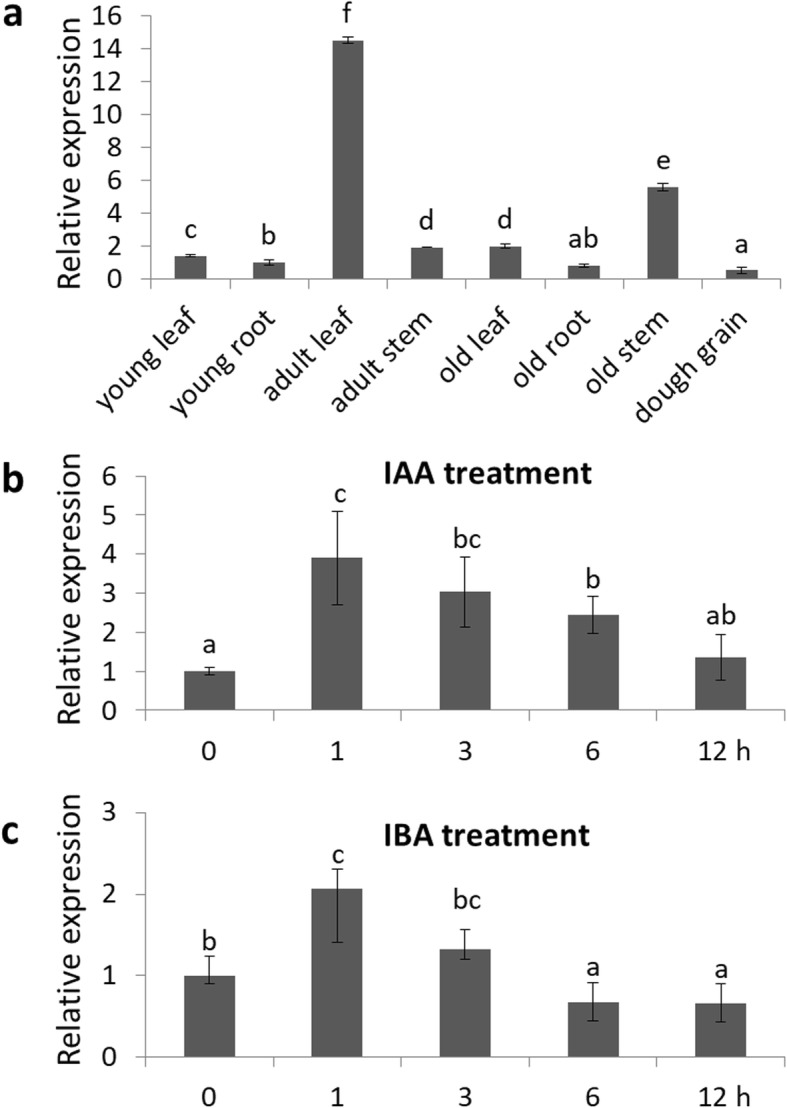
The expression pattern of *OsIAGT1*. **a** Expression pattern of *OsIAGT1* in rice. Young leaf and root were harvested from two-week-old seedlings. Adult leaf and stem were from plants growing for 2 months. Old leaf, root and stem were from plants growing for three and a half months. The dough grain was from maturing seeds. Transcript levels were normalized relatively to the mRNA levels of *OsActin1*. **c** and **d** Induction of *OsIAGT1* transcription by IAA or IBA. One-week-old wild-type rice was subjected to treatment with or without 10 μM IBA or 10 μM IAA for 1, 3, 6 and 12 h, respectively. Expression of *OsIAGT1* was analyzed with qRT-PCR analysis. The results represent mean ± SD from three replicates. Different lower-case letters indicate significant difference based on the Duncan’s multiple range test (*p* < 0.05)

We analyzed the tissue-specific expression pattern of *OsIAGT1* in rice with qRT-PCR (Fig. 5a). *OsIAGT1* expression is strongly activated in adult leaf and old stem, while in old root and dough grain *OsIAGT1* activity is lower than other part of rice tissue. These results indicated that *OsIAGT1* is predominantly expressed in the medium growth stage of rice and may play a role in adult leaf.

To investigate whether OsIAGT1 is involved in the auxin signaling, we treated wild-type rice with 10 μM IAA and 10 μM IBA, respectively, and the expression of *OsIAGT1* was determined by qRT-PCR analysis. Interestingly, *OsIAGT1* expression is significantly induced by both IAA and IBA treatments, and this induction already reached peak level after 1 h treatment (Fig. 5b and c). The auxin-induced *OsIAGT1* upregulation dropped gradually under longer treatment, dropping back to the original level after 12 h IAA treatment or even lower level after 6 h IBA treatment. The rapid *OsIAGT1* activation by auxin further supports the possible role of OsIAGT1 in modulating the auxin homeostasis and auxin signaling in rice.

### In Vivo Enzymatic Activity of OsIAGT1 towards Auxin

**Fig. 6:**
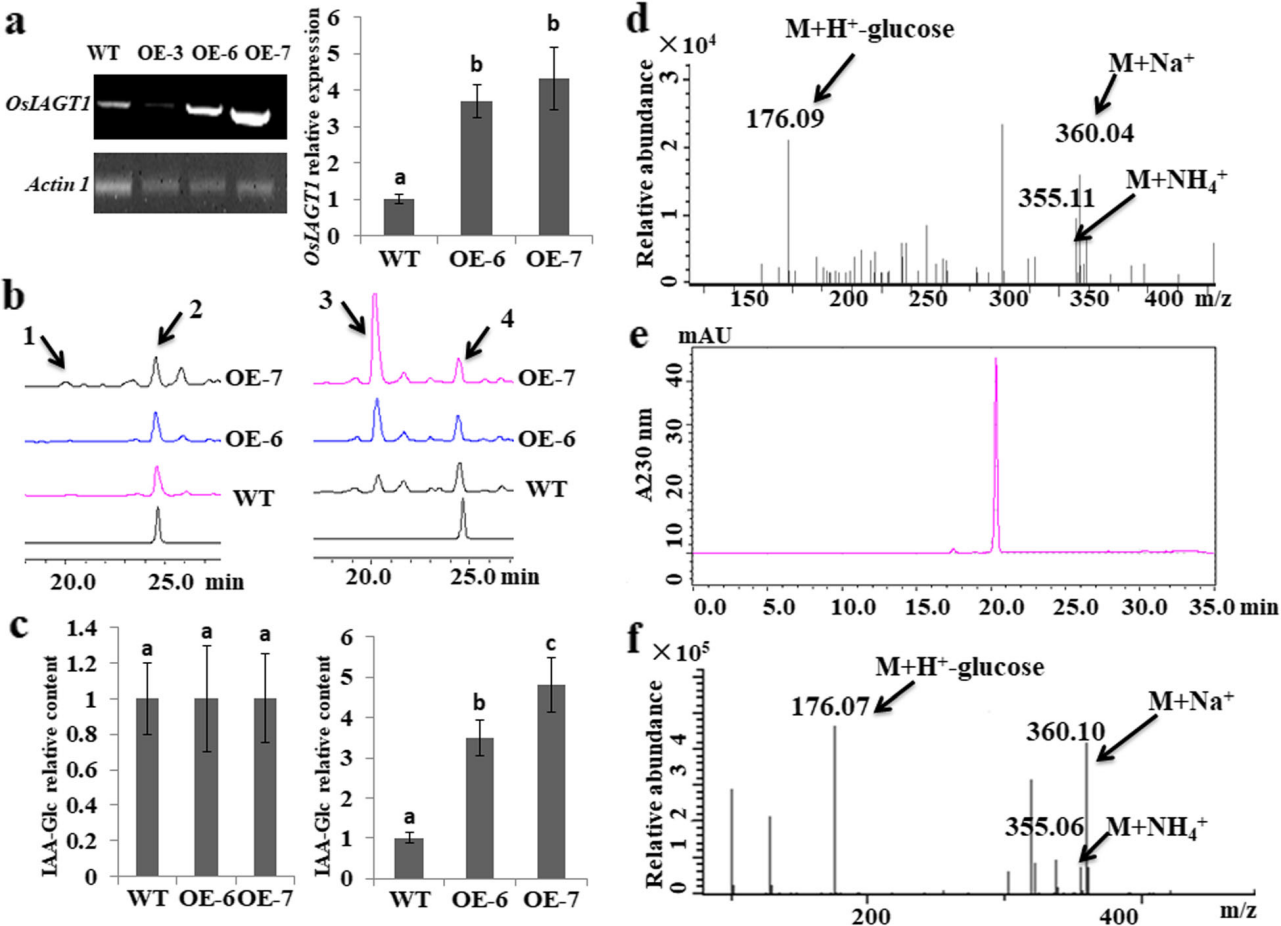
In vivo enzymatic activity of OsIAGT1 towards auxin. **a** Expression intensity of *OsIAGT1* OE lines. Left, RT-PCR analysis of *OsIAGT1* OE lines. Right, quantitative real-time PCR analysis of *OsIAGT1* in OE-6 and OE-7. Values are means ±SD (*n* ≥ 3). Different lower-case letters indicate significant difference based on the Duncan’s multiple range test (*p* < 0.05). **b** HPLC analysis of endogenous IAA-Glc in wild type and *OsIAGT1* OE lines. Left, no exogenous IAA was applied. Right, 100 μM IAA was applied for 12 h. 1 and 3 represent the presumable IAA-Glc; 2 and 4 represent the internal control (0.1 mM picloram) used in extraction process to monitor the recovery rate. **c** Relative content levels of endogenous IAA-Glc of wild type and OE lines. Left, no exogenous IAA was applied. Right, 100 μM IAA was applied for 12 h. Values are means ±SD (*n* ≥ 3). Different lower-case letters indicate significant difference based on the Duncan’s multiple range test (*p* < 0.05). **d** LC-MS/MS analysis of the endogenous IAA-Glc in rice. **e** Retention time of authentic standard IAA-Glc in HPLC analysis. **f** Ion peaks of authentic standard IAA-Glc in LC-MS/MS analysis under positive ion mode

In the light of auxin-induced upregulation of *OsIAGT1* and its in vitro activity towards auxins, we next investigated whether OsIAGT1 is capable of catalyzing auxin glucosylation in vivo. For this aim, the coding sequence of *OsIAGT1* was cloned and the overexpressing lines were created. The strong expression lines *OE-6* and *OE-7* were used for further analysis (Fig. 6a). Since endogenous IBA are barely detectable, the analysis was performed aiming to detect the glucose conjugates of IAA, a major form of endogenous auxins. IAA-Glc conjugates were extracted from one-week-old WT, *OE-6* and *OE-7* rice with or without a previous IAA inoculation process, and the level of presumable IAA-Glc conjugates was determined. As shown in Fig. 6b and c, the level of presumable IAA-Glc was only marginally higher than that in wild type plants (WT) due to a very low endogenous auxin level without a previous IAA inoculation. In contrast, after inoculation with 100 μM exogenous IAA, the level of the presumable IAA-Glc was strongly increased in all rice lines (peak 1 and peak 3), and the relative contents of IAA-Glc in overexpression lines were much higher than in WT. The peak 1 and 3 products were then analyzed with LC-MS (Fig. 6d). The result indicated a dominant ions m/z 176.09 (M + H^+^-glucose); m/z 355.11 (M + NH_4_^+^) and m/z 360.04 (M + Na^+^), fitting well with the molecular weight (MW) of IAA-Glc (MW = 337.00). In order to further verify whether the detected products were IAA-glucose conjugates, we synthesized the authentic IAA-Glc and performed the HPLC and LC-MS analyses. We found that the presumable IAA-Glc from both in vivo (Fig.6b, d) and in vitro (Fig.3a, c) has the same retention time in HPLC and same ion patterns in LC-MS analysis with the authentic standard IAA-Glc (Fig.6e, f). Therefore, we demonstrated that OsIAGT1 is able to catalyze the glucosylation of IAA to form corresponding glucose conjugate both in vivo and in vitro.

### Ectopic Expression of *OsIAGT1* Causes Reduced Auxin Content and Auxin Response

**Fig. 7:**
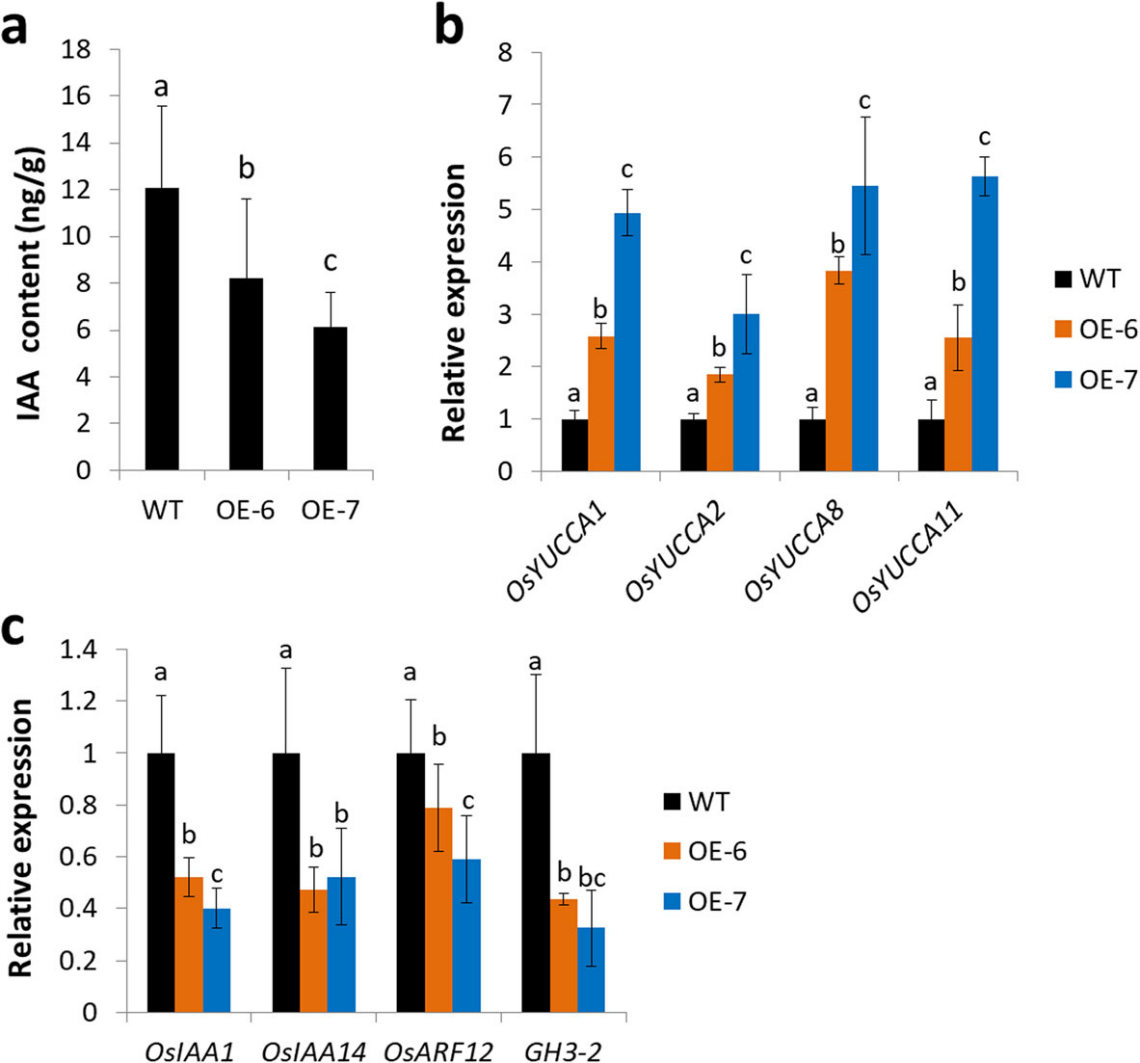
Auxin content and expression of auxin related genes in *OsIAGT1* overexpression rice plants. **a** IAA content in 7-day-old rice. **b** Expression of auxin synthesis genes in 7-day-old *OsIAGT1* overexpression rice plants compared to WT. **c** Expression of auxin-responsive genes in 7-day-old *OsIAGT1* overexpression rice plants compared to WT. Transcript levels were normalized relatively to the mRNA levels of *OsActin1*. Values are means ±SD (*n* ≥ 3). Different lower-case letters indicate significant difference based on the Duncan’s multiple range test (*p* < 0.05)

The activity of OsIAGT1 towards auxins implied that it might participate in regulating auxin-related growth in rice. To explore the possible role of OsIAGT1, firstly the endogenous IAA content of 7-day-old *OE-6* and *OE-7* rice plants were determined. The result showed that ectopic expression of *OsIAGT1* leads significantly reduced IAA content (Fig. 7a). Meanwhile, we also analyzed the endogenous IBA content because IBA is another favorite substrate of OsIAGT1 in vitro. However, we found that IBA was below the minimum detection limit in wild type and *OsIAGT1* overexpression lines under our detection conditions used, suggesting a very low concentration of naturally occurring IBA. Besides, we analyzed the expression of auxin-related genes in *OsIAGT1* overexpressing rice. In line with the reduced IAA content, the auxin biosynthetic genes *OsYUCCA1, OsYUCCA2, OsYUCCA8* and *OsYUCCA11*, which were known to be negatively regulated by auxin (Yamamoto et al. 2007), were strongly upregulated in *OsIAGT1* overexpressing lines (Fig. 7b), reflecting a metabolic compensation mechanism in plants. In addition, consistent with the reduced auxin content, those genes in auxin signaling pathway including the transcription repressor *OsIAA1* and *OsIAA14* (Tiwari et al. 2001; Jain et al. 2006), auxin-responsive *OsARF12* (Li et al. 2016) and auxin downstream gene *GH3–2* (Zhang et al. 2015) showed reduced expression in *OE-6* and *OE-7* rice plants (Fig. 7c). These results further confirmed that by glucosylation of auxin, OsIAGT1 plays an important role in regulating auxin content and activity.

### Ectopic Expression of *OsIAGT1* Leads to Reduced Shoot and Root Growth

**Fig. 8:**
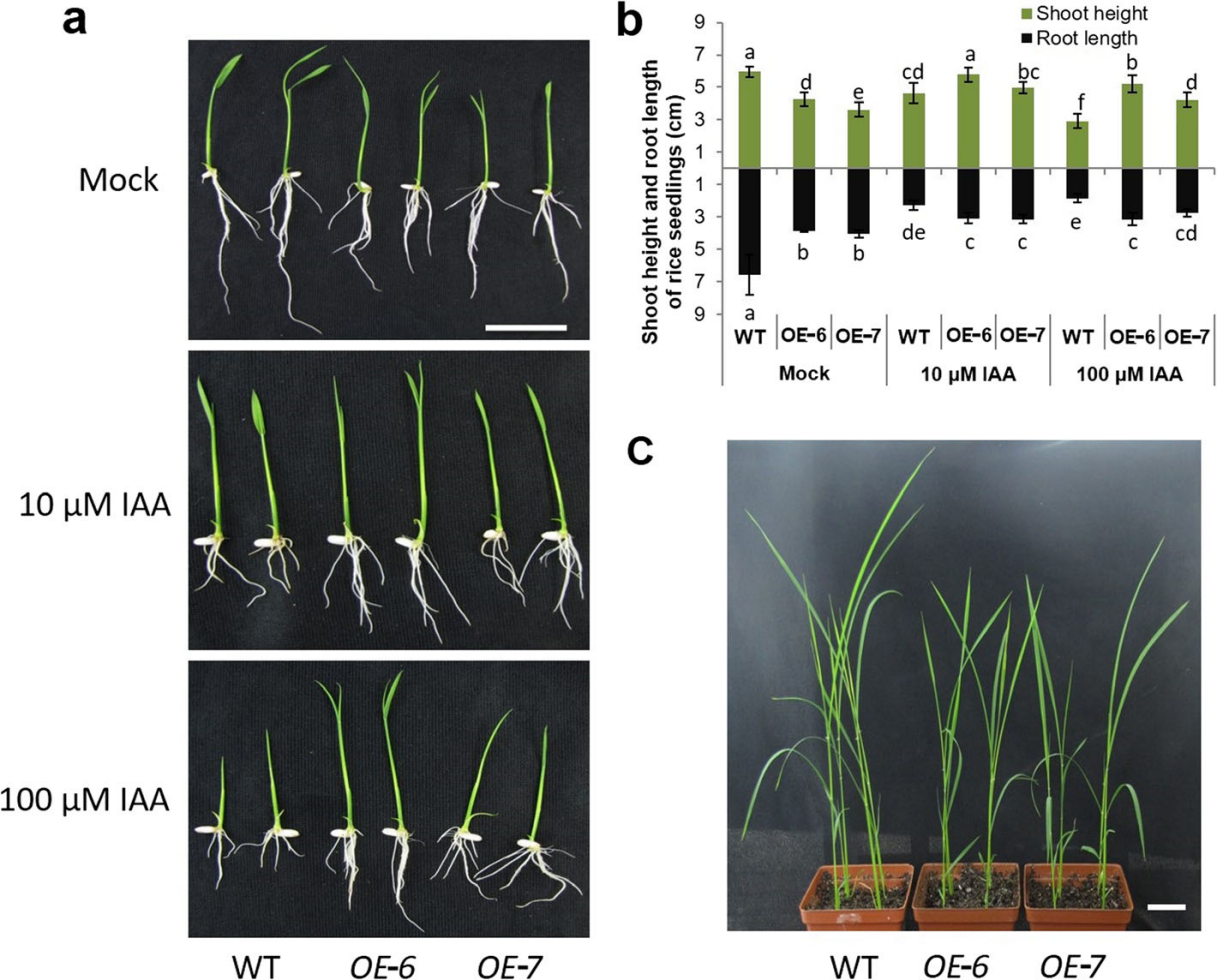
Effect of *OsIAGT1* overexpression on rice shoot and root growth. **a** 7-day-old WT, OE-6 and OE-7 rice seedlings before and after exogenous IAA treatment. Bars, 3 cm. **b** Statistic data of shoot height and root length of rice seedlings before and after IAA treatments. Values are means±SD (*n* ≥ 9). Different lower-case letters indicate significant difference based on the Duncan’s multiple range test (*p* < 0.05). **c** 3-week-old WT, OE-6 and OE-7 rice plants. Bar, 3 cm

Consistent with the reduced auxin content, we found that 7-day-old *OE-6* and *OE-7* seedlings showed reduced shoot height and root length compared with WT seedlings under non-treatment conditions (Fig. 8a and b). The reduced shoot stature was also observed in three-week-old *OsIAGT1* overexpressing plants (Fig. 8c). To further analyze the response of *OsIAGT1* overexpression lines to auxin, we treated rice seedlings with exogenous IAA. As shown in Fig. 8a and b, the treatment of 10 μM IAA appeared to inhibit root growth of WT seedlings, with much shorter root length than untreated WT seedlings. In contrast, *OE-6* and *OE-7* seedlings showed an increased shoot height and root elongation compared to WT seedlings under treatment. When being treated with excess amount IAA of 100 μM, WT seedlings showed obvious retarded growth, whereas *OE-6* and *OE-7* seedlings were more resistant to this excess IAA treatment likely due to the enhanced auxin glucosylation, leading to much better shoot and root growth than WT seedlings. Besides, we also examined whether the reduced growth observed in *OsIAGT1* overexpression plants under non-treatment condition can be rescued by lower concentration of IAA. As shown in Additional file 3: Figure S2, 1 μM IAA could restore the root growth of OE-6 and OE-7 rice seedlings, while shoot height was not largely changed. Combined with the results that *OsIAGT1* overexpression seedlings showed higher shoots and longer roots than WT under treatment with high concentration IAA (10 or 100 uM), our data suggest that OsIAGT1 is involved in regulating auxin level and auxin response by glucosylation, which might play an important role in mediating rice growth and development.

## Discussion

Auxin was known to regulate many aspects of plant growth and development. Due to the significance of auxin, the synthesis, transport and turnover of auxin are under tight control. Conjugating modification of auxin plays an important role in maintaining auxin homeostasis. In plants, glycosylation modification had been shown to mediate auxin homeostasis and auxin-related growth processes (Tognetti et al. 2010; Jin et al. 2013), yet in cereal crop rice, the responsible glycolsyltransferase towards auxin still remains largely unknown. In this study, we cloned the possible rice UGT genes that show high phylogenetic similarity to known auxin UGTs from maize and Arabidopsis, and the UGT gene with highest similarity, named as *OsIAGT1*, was expressed as recombinant protein. In vitro enzymatic analysis showed that OsIAGT1 is capable of catalyzing auxin glucosylation. OsIAGT1 is demonstrated to require a quite broad range of reaction conditions, with peak activity at 30 °С and pH 8.0. Interestingly, expression of *OsIAGT1* is auxin-induced in rice, implying that OsIAGT1 might be responsible for in vivo auxin glucosylation. This is verified by enhanced formation of IAA glucose conjugates in *OsIAGT1* overexpression lines. Moreover, in these overexpression lines the auxin content is significantly reduced, leading to the upregulation of auxin synthesis genes and downregulation of auxin responsive genes. Possibly due to reduced auxin response, *OsIAGT1* overexpression lines showed reduced shoot height and root length. As a complement to the recent report on the same UGT protein OsIAAGLU (Yu et al. 2019), our results confirmed that OsIAGT1 /OsIAAGLU is capable of catalyzing auxin glucosylation in rice cells, and further strengthened the role of OsIAGT1/OsIAAGLU in mediating auxin homeostasis and thus regulating auxin-related rice growth.

Tissue expression pattern analysis showed that *OsIAGT1* expression is high in adult tissues including adult leaf and old stem, where OsIAGT1 might function in transforming auxin to inactive glucose ester conjugate form. On the other hand, in younger tissues *OsIAGT1* expression is quite low. This is consistent with the fact that younger tissues accumulate active auxin to promote plant growth and development (Scarpella et al. 2010). Moreover, it is also reflected in our result that ectopic expression of *OsIAGT1* leads shortened shoot and root (Fig. 8).

By in vitro enzymatic analysis, OsIAGT1-GST recombinant protein is demonstrated to have a broad range of reaction conditions, which implied that OsIAGT1 might play a role when plants are facing unfavorable conditions. This is reminiscent of the role of other stress response-related UGT proteins, e.g. Arabidopsis UGT79B2 and UGT79B3 are capable of promoting cold stress resistance by glycosylation of anthocyanins (Li et al. 2017). Similarly, maize glycosyltransferase UFGT2 modifies flavonols and contributes to plant acclimation to abiotic stresses (Li et al. 2018). Moreover, it had been shown that auxin conjugates are usually inactive forms (Ludwig-Müller 2011). Therefore, OsIAGT1 might also contribute to plant stress response, in which it transforms auxin to inactive glucose conjugates when facing stress conditions. It would be interesting to further analyze the role of OsIAGT1 in stress responses in future studies.

## Materials and Methods

### Chemicals

Most of the substrates used in this study were purchased from Sigma-Aldrich (St. Louis, MO USA). UDP-Glucose was purchased from Meryer (Shanghai, China). Glutathione-coupled Sepharose 4B beads and reduced form glutathione were obtained from Amersham Pharmacia (Piscataway, NJ USA). Restriction enzymes, ligation enzymes and PrimeSTAR HS DNA Ploymerase were purchased from TaKaRa (Shiga, Japan). Authentic IAA-glucose was synthesized by a sugar-biotech company (Chemily Glycoscience Co., GA, USA).

### Phylogenetic Analysis

BLAST searches in rice genome databases (the Rice Annotation Project Database [RAP-DB; http://rapdb.dna.affrc.go.jp/] and the Rice Genome Annotation Project [http://rice.plantbiology.msu.edu/]) web sites were carried out using the deduced amino acid sequences of maize (*Zea mays*) UGT iaglu (L34847) and Arabidopsis UGT84B1, UGT84B2, UGT74E2 and UGT74D1 as queries, which were revealed to encode auxin UGTs. Twelve putative glycosyltransferases of rice were included in this tree construct. The phylogenetic tree was constructed by the neighbor-joining method with a bootstrap test using MEGA 4.0.2 software (http://www.megasoftware.net/).

### Cloning and Plasmid Construction of Putative Auxin Glucosyltransferases of Rice

Full-length cDNA of putative auxin glucosyltransferases of rice were amplified from a Japonica rice cultivar (*Oryza sativa*, Nipponbare) by reverse transcription-PCR (RT-PCR) method using gene-specific primers. Then, each gene was subcloned into prokaryotic expression plasmid using standard molecular biology techniques. *OsIAGT1* (LOC_Os03g48740) was cloned by PCR-based splicing and overlap extension method of RT-PCR with two pair of primers, *OsIAGT1*-P1: 5′-GCCGGATCCATGGCGCATGT-3′; *OsIAGT1*-P2: 5′-GCCCTCGAGTCACATCTCCGACGCTGC-3′; *OsIAGT1*-ex1: 5′-GGGTCGACAACCCAGCCAAAACCTCGCTCTCCAGCTCGTCGAACGAGT-3′; *OsIAGT1*-ex2: 5′-TGGAGAGCGAGGTTTTGGCTGGGTTGTCGACCC-3′. P1 and ex1 were used to get the first exon, P2 and ex2 to get the second exon. Then P1 and P2 were merged to get full cDNA. *OsIAGT1*-P1 and *OsIAGT1*-P2 contain restriction sites at 5′ terminals for BamHI and XhoI, respectively. The PCR product was cloned by recombination into pBluescriptSK. In order to obtain a prokaryotic expression vector with suitable and multiple restriction sites, pGEX-2 T vector was modified for restriction endonuclease sites BamHI, NdeI, NotI, SphI, NcoI, SalI, SacI, XhoI, HindIII, EcoRI and is designated as pGEX-3H. The cDNA of *OsIAGT1* were subcloned into pGEX-3H with BamHI and XhoI to obtain the expression plasmid of glutathione S-transferase (GST)-OsIAGT1 fusion protein. Meanwhile, the cDNA of UGT74D1, an Arabidopsis gene encoding auxin gluocsyltransferase, was also subcloned into pGEX-3H to obtain the expression plasmid of GST-UGT74D1 fusion protein used as positive control in this study.

### Protein Purification and Enzyme Activity Assay

*Escherichia coli* strain XL1-Blue carrying the expression plasmid of fusion construct was used to produce the fusion protein. Soluble recombinant protein was induced and purified as previously described (Hou et al. 2004). Protein concentration of the eluted fractions was determined with Coomassie Protein Assay Reagent (Thermo Scientific) using bovine serum albumin as reference. The purified recombinant fusion protein was also analyzed by SDS-PAGE as described (Green 2012). The glycosyltransferase activity assay was carried out following a previously described protocol (Tognetti et al. 2010). Briefly, the assay mix (100 μl) contained 2 μg of purified fusion protein (2 μg of purified GST protein as control), 5 mM UDP-glucose, 1 mM plant hormone, 50 mM Tris.HCl (pH 7.0), 2.5 mM MgSO4, 10 mM KCl and 14.4 mM 2-mercaptoethanol. The reactions were carried out at 30°С for 3 h and then stopped by the addition of 10 ml of trichloroacetic acid (240 mg/ml), quick-frozen, and stored at − 20°С before reverse-phase high performance liquid chromatography (HPLC) analysis.

### HPLC and LC-MS Analysis

Reversed-phase HPLC was carried out on a Shimadzu HPLC system (Japan). Twenty μl of each sample was loaded by means of an auto-sampler SIL-20A onto a 5 μm C18 column (150 mm × 4.6 mm; Welch, Ultimate). A linear gradient with increasing methanol (solvent A) against distilled H_2_O (solvent B) at a flow rate of 1 ml/min over 40 min was used to separate the glucose conjugates from their aglycones. Both solutions contained 0.1% H_3_PO_4_. Each peak on the chromatogram was monitored between 190 nm and 430 nm.

The products of auxin conjugates synthesized by recombinant OsIAGT1 were further confirmed by the liquid chromatograph mass spectrometry (LC-MS) system (Thermo Scientific) including the Surveyor autosampler and MS pump (Thermo-Finnigan, San Jose, CA, USA). The methods and mobile phases were similar to HPLC condition except that 0.1% acetic acid instead of 0.1% H_3_PO_4_. The mass spectrometer operated in a positive electrospray ionization mode with 30 eV and a probe voltage of 3.0 kV. The temperature was set to 350 °С. The data acquisition and analysis were performed with Xcalibur software (version 2.0.6).

### Factors Affecting the Activity of Recombinant OsIAGT1

Because IAA is the major auxin in plants, we choose the IAA in this study as substrate for analyzing the factors affecting the enzyme activity. The calculation of enzyme activity was based on the reduced numbers of moles according to peak area of the substrate before and after reaction. Factors tested include temperature, buffer type and pH. All the reaction mix (100 μl) contained 0.2 μg of recombinant OsIAGT1, 5 mM UDP-glucose, 1 mM IAA, 2.5 mM MgSO_4_, 10 mM KCl, 14.4 mM 2-mercaptoethanol. For the temperature test, 50 mM Tris.HCl (pH 7.0) was added and the reactions were performed at five different temperature points (20°С, 25°С, 30°С, 35°С and 40°С) for 30 min. For the buffer and pH test, 50 mM Tris buffer (pH 7.0–9.0), 100 mM Phosphate buffer (pH 6.0–8.0), 50 mM MES buffer (pH 6.0–7.0) was added and the reactions were performed at 30°С for 30 min. All the reactions were stopped by adding 10 μl trichloroacetic acid (240 mg/ml), quick-frozen, and stored at − 20°С before reverse-phase HPLC analysis.

For the calculation of relative enzyme activity of OsIAGT1 towards different forms of auxins, we calculated the peak area of the substrates before and after reaction. The relative enzyme activity was defined as the percent that the reduced peak area after reaction accounts for relative to that before reaction.

### *OsIAGT1* Expression Pattern Analysis

To analyze the profiling of *OsIAGT1* expression in different tissues, young leaf and root were harvested from two-week-old seedlings. Adult leaf and stem were from plants growing for 2 months. Old leaf, root and stem were from plants growing for three and a half months. The dough grain was from maturing seeds. The corresponding materials were collected and immediately frozen in liquid nitrogen and total RNA was isolated to detect the relative expression in different tissues.

To detect the expression change of *OsIAGT1* by auxin treatment, one-week-old wild-type rice seedlings were treated with 10 μM of IAA or IBA for 1, 3, 6 and 12 h, respectively. Then the seedlings were collected for RNA extraction and Quantitative Real-time PCR (qRT-PCR) analysis.

To detect the transcription levels of auxin related genes, one-week-old seedlings of transgenic plants and wild type plants were collected for RNA extraction and qRT-PCR analysis.

### Generation of *OsIAGT1*-Overexpressing Transgenic Rices

For creating *OsIAGT1* overexpression lines, the full-length *OsIAGT1* coding sequence was amplified by PCR from the sub-cloned prokaryotic expression plasmid mentioned above using gene-specific primers, and then introduced into the PUN1301 vector under the Ubiquitin promoter (PUN1301 is a pCambia1301-derived plasmid with the Ubiquitin promoter inserted in multiple cloning sites, and the hygromycin resistance used for selection marker). This vector construct was introduced into *A. tumefaciens* strain EHA105 for transformation of wild type rice (Nipponbare). T2 homozygous *OsIAGT1* overexpression lines were used for further analyses.

### Glycosylated Metabolites of Auxin in Transgenic Rice

For analyzing the amount of the auxin glucose conjugates of in the transgenic rice, the wild type and overexpression lines (line 6, line 7) were grown for 7 days, and moved to distilled water with or without 100 μM IAA. After incubation for 12 h, plant tissues from each line was collected and frozen in liquid nitrogen prior to the extraction. The extraction of IAA glucose conjugates was carried out following the method described by Hou et al. (2004). The last supernatant was collected and analyzed with HPLC. 0.1 mM picloram was added as internal control to monitor the recovery rate. The relative amounts of IAA glucose conjugates of different transgenic lines can be calculated from the height of peak of HPLC. The putative IAA glucose conjugate was identified by LC–MS and using the authentic standard IAA-glucose as control.

### IAA Concentration Measurement

The sample preparation, extract analysis, IAA identification, and IAA quantification were performed at Wuhan MetWare Biotechnology Co., Ltd. (www.metware.cn), following their standard procedures. IAA concentration of 7-day-old WT, *OE-6* and *OE-7* rice plants was measured using an LC-ESI-MS/MS system (HPLC, Shim-pack UFLC SHIMADZU CBM30A system; MS, Applied Biosystems 6500 Triple Quadrupole). In brief, fresh plant materials (50 mg) were frozen in liquid nitrogen, ground into powder, and extracted with 0.5 ml methanol/water/formic acid (15:4:1,V/V/V) at 4 °C. The extract was vortexed (10 min) and centrifuged at 14,000 rpm under 4 °C for 5 min. The supernatants were collected, repeat the steps above, vortexed (5 min) and centrifuged (5 min). The combined extracts were evaporated to dryness under nitrogen gas stream, reconstituted in 80% methanol (V/V), ultraphoniced (1 min) and filtrated (PTFE, 0.22 μm; Anpel). Then the sample extracts were analyzed using the LC-ESI-MS/MS system and the analytical conditions were as follows. HPLC: column, Waters ACQUITY UPLC HSS T3 C18 (1.8 μm, 2.1 mm*100 mm); solvent system, water (0.04% acetic acid): acetonitrile (0.04% acetic acid); gradient program, 90: 10 V/V at 0 min, 40: 60 V/V at 5 min, 40: 60 V/V at 7 min, 90:10 V/V at 7 min, 90:10 V/V at 10 min; flow rate, 0.35 ml/min; temperature, 40 °C; injection volume: 2 μl. The effluent was alternatively connected to the API 6500 Q TRAP LC/MS/MS System, equipped with an ESI Turbo Ion-Spray interface, operating in positive ion mode and controlled by Analyst 1.6 software (AB Sciex). The ESI source operation parameters were as follows: ion source, turbo spray; source temperature 500 °C; ion spray voltage (IS) 4500 V; curtain gas (CUR) were set at 35.0 psi; the collision gas (CAD) was medium. DP and CE for individual MRM transitions were done with further DP and CE optimization. A specific set of MRM transitions were monitored for each period according to the plant hormones eluted within this period. Endogenous IAA was monitored by the transition of *m/z* 176.1 → *m/z* 130.1.

By measuring the peak area of a series of standard concentration of diluted IAA solution (ranging from 0.02 ng/ml to 2000 ng/ml), a linear equation of the standard curve was calculated. With the determined peak area of IAA the absolute IAA content in plants (ng/g) was calculated.

### Quantitative RT-PCR

For all qRT-PCR analyses in this study, reverse transcription reactions were performed with the PrimeScript RT reagent kit with gDNA Eraser (Takara, Japan). qRT-PCR reactions were carried out on real-time thermal cycling system (Bio-Rad, USA), and the SYBR-Green Ex Taq II kit (TaKaRa, Japan) was used for detecting gene expression abundances. qPCR signals were normalized to *OsActin1*. All reactions were performed with at least three replicates. Primers used in this study were provided in Additional file 1: Table S1.

### RAP-DB Accession Numbers

*OsIAGT1 (Os03g0693600), OsYUCCA1 (Os01g0645400), OsYUCCA2 (Os05g0528600), OsYUCCA8 (Os03g0162000), OsYUCCA11 (Os12g0189500), OsAct1 (Os03g0718100), GH3–2 (Os01g0764800), OsIAA14 (Os03g0797800), OsARF12 (Os04g0671900).*

## Supplementary information

**Additional file 1: Table S1.** Primers used for quantitative PCR in gene expression analysis.

**Additional file 2: Figure S1.** HPLC and LC-MS analysis of reaction products from IPA (a), NAA (b), 2,4-D (c) and ICA (d) catalyzed by OsIAGT1. The left graph shows the HPLC analysis and the right shows the LC-MS analysis. 1 and 2 denote the reactions with recombinant protein OsIAGT1 and GST (negative control), respectively. “A” denotes the possible products; “B” denotes the substrate.

**Additional file 3: Figure S2.** Effects of *OsIAGT1* overexpression on rice shoot and root growth. **a** 7-day-old WT, OE-6 and OE-7 rice seedlings before and after exogenous treatment with 1 μM IAA. Bar, 3 cm. **b** Shoot height and root length of rice seedlings before and after IAA treatments. Values are means ±SD (*n* ≥ 9). Different lower-case letters indicate significant difference based on the Duncan’s multiple range test (*p* < 0.05).

## Data Availability

All data generated or analysed during this study are included in this article and its supplementary information files.

## Abbreviations

2,4-D: 2,4-dichlorophenoxyacetic acid
4-Cl-IAA: 4-chloroindole-3-acetic acid
GFP: Green fluorescent protein
GST: Glutathione S-transferase
GT: Glucosyltransferase
HPLC: High performance liquid chromatography
IAA: Indole-3-acetic acid
IBA: Indole-3-butyric acid
IPA: Indole-3-propionic acid
LC-MS: Liquid chromatograph mass spectrometry
NAA: Naphthalene-1-acetic acid
PAA: Phenylacetic acid
qRT-PCR: Quantitative real-time PCR
RT-PCR: Reverse transcription-PCR
UGT: UDP-glycosyltransferase
