# Supplementary material for: OsIAGT1 Is a Glucosyltransferase Gene Involved in the Glucose Conjugation of Auxins in Rice
Source: Rice (N Y). 2019 Dec 18;12:92. doi: 10.1186/s12284-019-0357-z (PMC6920275; doi:10.1186/s12284-019-0357-z)
Supplement: Supplementary file 1 — Additional file 1: Table S1. Primers used for quantitative PCR in gene expression analysis. [file 12284_2019_357_MOESM1_ESM.docx]

**Table S1. Primers used for quantitative PCR in gene expression analysis**

| Primer | Sequence (5’-3’) |
| --- | --- |
| qOsIAGT1F | GACCAGCCCACCAACGCCCTGC |
| qOsIAGT1R | CAAACCAGCGTCTTCCACTTCT |
| qOsYUCCA1F | TCATCGGACGCCCTCAACGTCGC |
| qOsYUCCA1R | GGCAGAGCAAGATTATCAGTC |
| qOsYUCCA2F | GTCCAAAGGGAGGAGTCGTCCAG |
| qOsYUCCA2R | GCATGATGTTTACACCCGGCCTT |
| qOsYUCCA8F | CCAACATCTCCTCGGTGTAG |
| qOsYUCCA8R | GCATCAGACAAGCAACATCC |
| qOsYUCCA11F | ATGCCCAAGAAGGACTTCCC |
| qOsYUCCA11R | GAAGGCCTTGACGTCATTAGCA |
| qOsIAA14F | CCGTCGCCTATGAGGACAAG |
| qOsIAA14R | CGCATTATCCGCAGCTTCTT |
| qOsARF12F | TCTCGATTTAATCAGCCTGT |
| qOsARF12R | CAACCATACATTGCATTCTG |
| qGH3-2F | TCATGCCCGTCATGAACTTG |
| qGH3-2R | TCGTCTCCGACTTGATGAACAG |
| qOsActin1F | CGGGAAATTGTGAGGGACAT |
| qOsActin1R | AGGAAGGCTGGAAGAGGACC |
