# Supplementary material for: OsIAGT1 Is a Glucosyltransferase Gene Involved in the Glucose Conjugation of Auxins in Rice
Source: Rice (N Y). 2019 Dec 18;12:92. doi: 10.1186/s12284-019-0357-z (PMC6920275; doi:10.1186/s12284-019-0357-z)
Supplement: Supplementary file 2 — Additional file 2: Figure S1. HPLC and LC-MS analysis of reaction products from IPA (a), NAA (b), 2,4-D (c) and ICA (d) catalyzed by OsIAGT1. The left graph shows the HPLC analysis and the right shows the LC-MS analysis. 1 and 2 denote the reactions with recombinant protein OsIAGT1 and GST (negative control), respectively. “A” denotes the possible products; “B” denotes the substrate. [file 12284_2019_357_MOESM2_ESM.docx]

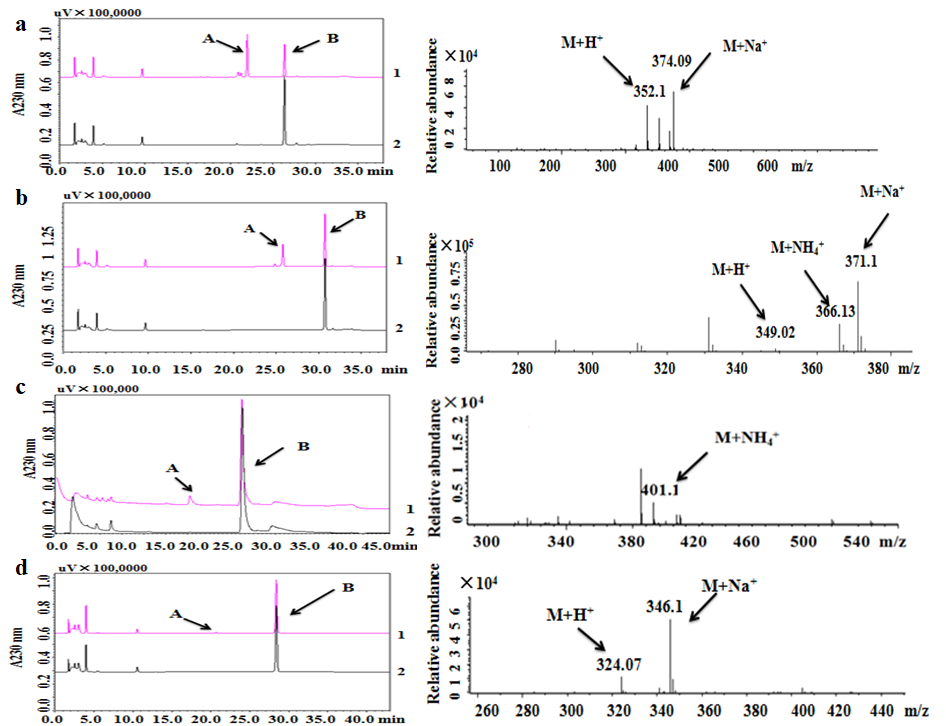


**Figure S1.** HPLC and LC-MS analysis of reaction products from IPA (a), NAA (b), 2,4-D (c) and ICA (d) catalyzed by OsIAGT1. The left graph shows the HPLC analysis and the right shows the LC-MS analysis. 1 and 2 denote the reactions with recombinant protein OsIAGT1 and GST (negative control), respectively. “A” denotes the possible products; “B” denotes the substrate.
