# Supplementary material for: OsIAGT1 Is a Glucosyltransferase Gene Involved in the Glucose Conjugation of Auxins in Rice
Source: Rice (N Y). 2019 Dec 18;12:92. doi: 10.1186/s12284-019-0357-z (PMC6920275; doi:10.1186/s12284-019-0357-z)
Supplement: Supplementary file 3 — Additional file 3: Figure S2. Effects of OsIAGT1 overexpression on rice shoot and root growth. a 7-day-old WT, OE-6 and OE-7 rice seedlings before and after exogenous treatment with 1 μM IAA. Bar, 3 cm. b Shoot height and root length of rice seedlings before and after IAA treatments. Values are means ±SD (n ≥ 9). Different lower-case letters indicate significant difference based on the Duncan’s multiple range test (p < 0.05). [file 12284_2019_357_MOESM3_ESM.docx]

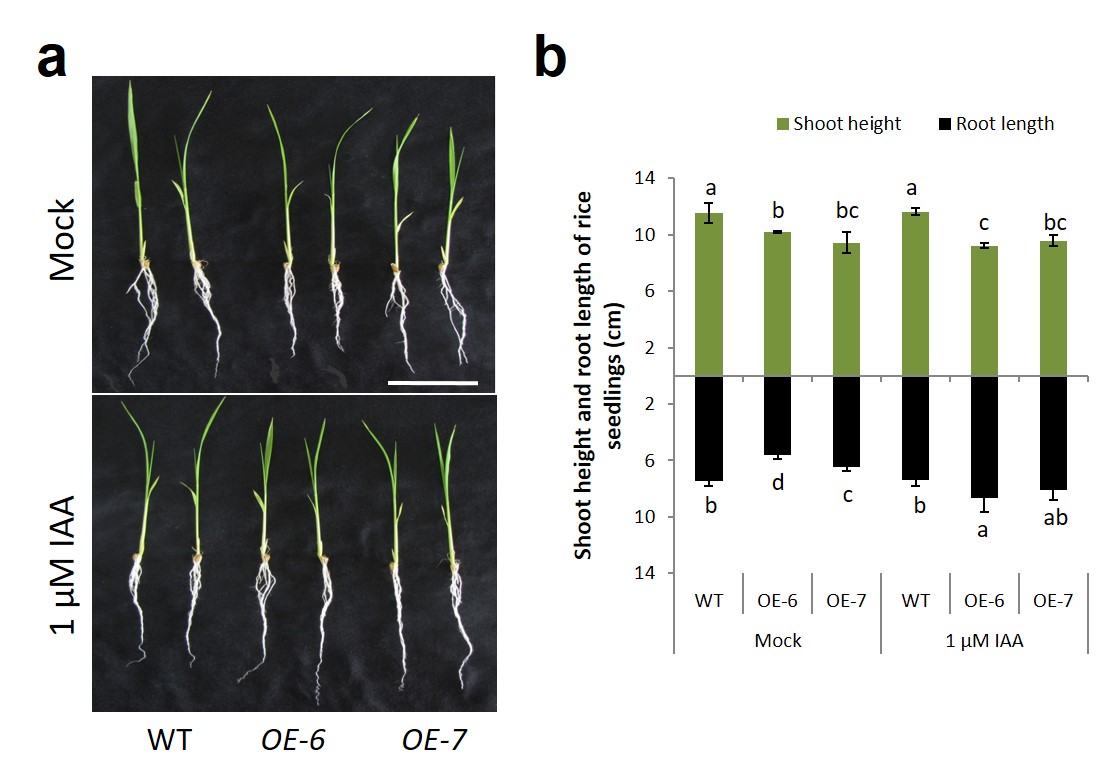


**Figure S2.** Effects of *OsIAGT1* overexpression on rice shoot and root growth.

**a** 7-day-old WT, OE-6 and OE-7 rice seedlings before and after exogenous treatment with 1 μM IAA. Bar, 3 cm. **b** Shoot height and root length of rice seedlings before and after IAA treatments. Values are means ±SD (n≥9). Different lower-case letters indicate significant difference based on the Duncan’s multiple range test (p < 0.05).
